# Deciphering the Taxonomic Delimitation of *Ottelia acuminata* (Hydrocharitaceae) Using Complete Plastomes as Super-Barcodes

**DOI:** 10.3389/fpls.2021.681270

**Published:** 2021-07-15

**Authors:** Yunheng Ji, Jin Yang, Jacob B. Landis, Shuying Wang, Zhenyan Yang, Yonghong Zhang

**Affiliations:** ^1^CAS Key Laboratory for Plant Diversity and Biogeography of East Asia, Kunming Institute of Botany, Chinese Academy of Sciences, Kunming, China; ^2^Yunnan Key Laboratory for Integrative Conservation of Plant Species with Extremely Small Populations, Kunming Institute of Botany, Chinese Academy of Sciences, Kunming, China; ^3^School of Life Sciences, Yunnan University, Kunming, China; ^4^School of Integrative Plant Science, Section of Plant Biology and the L.H. Bailey Hortorium, Cornell University, Ithaca, NY, United States; ^5^School of Life Sciences, Yunnan Normal University, Kunming, China

**Keywords:** species boundary, molecular identification, DNA barcoding, taxonomy, threatened species, aquatic plant

## Abstract

Accurate species delimitation and identification, which is a challenging task in traditional morphology-based taxonomy, is crucial to species conservation. *Ottelia acuminata* (Hydrocharitaceae) is a severely threatened submerged macrophyte endemic to southwestern China. The taxonomy of *O. acuminata*, which has long been in dispute, remains unresolved, impeding effective conservation and management practices. Here, we aim to address the long-standing issues concerning species boundary and intraspecific subdivision of *O. acuminata* using complete plastome sequences as super-barcodes. The taxonomic delimitation of *O. acuminata* was explored using phylogenetic inference and two independent sequence-based species delimitation schemes: automatic barcode gap discovery (ABGD) and multi-rate Poisson tree processes (mPTP). The reciprocally reinforcing results support the reduction of the closely related congeneric species, *O. balansae* and *O. guanyangensis*, as two conspecific varieties of *O. acuminata*. Within the newly defined *O. acuminata*, accurate varietal identification can be achieved using plastome super-barcodes. These findings will help inform future decisions regarding conservation, management and restoration of *O. acuminata*. This case study suggests that the use of plastome super-barcodes can provide a solution for species delimitation and identification in taxonomically difficult plant taxa, thus providing great potential to lessen the challenges of inventorying biodiversity, as well as biologically monitoring and assessing threatened species.

## Introduction

Species are a fundamental unit of biodiversity (Claridge et al., 1997). Estimating how many species are under threat is an essential step in setting conservation priorities (May, 1988; May and Beverton, 1990; Margules and Pressey, 2000; Dirzo and Raven, 2003; Mace et al., 2003). Given that prioritizing species for natural conservation heavily depends on reliable species identification, often requiring prior species delimitation (Mace, 2004), accurate delineation of species boundaries is crucial for species conservation (Rojas, 1992; Sites and Crandall, 1997; Prance et al., 2000; Mace, 2004). Nevertheless, species delimitation is a challenging task in numerous plant taxa due to a lack of taxonomically robust morphologies, especially given that the majority of plant species are recognized based on morphological differences alone which can fail to discriminate cryptic species (Duminil and Di Michele, 2009). Recent general acceptance suggests that species should be recognized as evolutionarily distinct entities possessing significant morphological and evolutionary distinctiveness, or niche differentiation (de Queiroz, 1998; Wiley and Mayden, 2000; Sites and Marshall, 2003). This acceptance drove the development of a multidisciplinary approach that utilizes morphological, genetic, ecological, and even metabolomic data in discriminating species (Sites and Marshall, 2003; Duminil and Di Michele, 2009; Su et al., 2015; Eisenring et al., 2016; Cheng et al., 2020).

Analysis of DNA sequence variation can provide useful genetic information to develop robust species delimitation for the purpose of conservation and utilization (Hebert et al., 2003; Duminil et al., 2012; Puillandre et al., 2012b). DNA barcoding, a technique that involves the standardized use of one or a few DNA regions (DNA barcodes) for identification and discrimination of species (Hebert et al., 2003; Kress et al., 2005; Hollingsworth, 2011; Hollingsworth et al., 2011, 2016), has proven useful in facilitating species delimitation (e.g., Duminil et al., 2012; Puillandre et al., 2012a; Kekkonen and Hebert, 2014; Mutanen et al., 2015; Hausmann et al., 2016). Nevertheless, the efficacy of standard DNA barcodes (i.e., *rbcL*, *matK*, *trnH-psbA*, and ITS) in either identification or delimitation of plant species remains problematic, especially in recently diverged or rapidly radiating taxa (Hollingsworth et al., 2009, 2011, 2016; Hollingsworth, 2011; Coissac et al., 2016). Benefiting from the development and advancement of next-generation DNA sequencing (NGS) technologies, genome-wide sequence data are increasingly used as extended DNA barcodes for species identification and delimitation, providing a possible solution for credibly delineating species boundaries in plants, especially in taxonomically perplexing taxa (Coissac et al., 2016; Hollingsworth et al., 2016).

The complete plastid genomes (plastomes) possess more variable loci by orders of magnitude than standard DNA barcodes and thus have great potential to improve resolution in species discrimination (Nock et al., 2011; Kane et al., 2012; Ruhsam et al., 2015). Additionally, they are highly repetitive genome components in each plant cell, making plastome assembly feasible via a relatively shallow sequencing depth (Straub et al., 2012). Due to these advantages, complete plastome DNA sequences have been recommended for consideration as “super-barcodes” for plant species discrimination and delimitation (Dodsworth, 2015; Li et al., 2015). Several recent studies attempted to use plastome super-barcodes to decipher species boundaries in a wide spectrum of plant lineages (e.g., Firetti et al., 2017; Ji et al., 2019, 2020; Zhu et al., 2019; Li L. et al., 2020; Ślipiko et al., 2020). However, most of the studies inferred tree topology solely under the premise of reciprocal monophyly to explore species boundaries, thus likely producing biased delimitation schemes. In view of this, empirical studies that employ multiple delimitation methods are urgently needed to evaluate the usefulness of plastome super-barcodes in species delimitation especially for conservation and management purposes.

*Ottelia* Persoon (Hydrocharitaceae) is a pantropic genus with ∼21 species of submerged macrophytes primarily distributed in tropic Africa and southeast Asia, according to the most comprehensive taxonomic revision of the genus (Cook et al., 1983). However, recent studies based on DNA sequence data reveal that two widespread species within the genus, namely *Ottelia alismoides* (Ito et al., 2019) and *O. ulvifolia* (Li et al., 2020b), contain cryptic species, suggesting that the alpha taxonomy of *Ottelia* remains ambiguous. Given that the majority of species within the genus are threatened with local or global extinction (Phillips et al., 2016; Zhang et al., 2017), a credible taxonomy is necessary for conservation and management of extant *Ottelia* species.

*Ottelia acuminata*, a severely threatened submerged macrophyte, consisting of six phenotypic varieties, is an endemic species occurring in freshwater lakes, ponds and rivers in Southwest China (Jiang et al., 2005; Wang et al., 2010). This species is categorized as vulnerable (VU) under the criteria “A2c” in the China Species Red List (Qin et al., 2017), and many wild populations have deteriorated or even perished during the past 30 years due to habitat degradation, anthropogenic disturbances, and introduction of herbivorous fish (Li, 1985, 1988; Godo et al., 2003; Liang and Li, 2007; Jiang et al., 2010; Yang et al., 2012). Remarkably, the taxonomic delimitation of *O. acuminata* as well as its varieties (*O. acuminata* var. *acuminata*, *O. acuminata* var. *crispa*, *O. acuminata* var. *jingxiensis*, *O. acuminata* var. *lunanensis*, *O. acuminata* var. *songmingensis*, and *O. acuminata* var. *tonghaiensis*) remains controversial. For instance, *O. acuminata* var. *crispa* recognized by Li (1981) was treated as a separate species, *O. crispa*, by Dandy (1935) and Wang (1986). *Ottelia acuminata* var. *lunanensis* was accepted as a variety by Li (1981) and Wang et al. (2010), while Cook et al. (1983) reduced it to a synonym of *O. acuminata* var. *acuminata.* Despite *O. acuminata* var. *tonghaiensis* being described as a variety by Li (1981), Wang et al. (2010) combined it with *O. acuminata* var. *acuminta.* Additionally, genus-level phylogenetic analyses fail to resolve *O. acuminata* as a monophyletic unit (Li et al., 2020c). Therefore, the taxonomic delimitation of *O. acuminata* and its phenotypic varieties needs to be re-evaluated.

In this study, we aim to clarify the long-standing controversies in species delimitation and intraspecific subdivision of *O. acuminata* using plastome super-barcodes. We employed a genome skimming approach (Straub et al., 2012) to generate complete plastome DNA sequences as well as sampling multiple accessions of each varieties within the species. Under a phylogenetic framework, we first examined whether *O. acuminata* and the phenotypic varieties are monophyletic entities. Next, we used two independent molecular species delimitation methods to explore the species boundaries of *O. acuminata* and closely related species. The results suggest that the species boundary of *O. acuminata* should be expanded to accommodate *O. balansae* and *O. guanyangensis* as two conspecific varieties. The newly circumscribed *O. acuminata*, including the conspecific varieties, can be distinguished with plastome super-barcodes. The findings will help inform future decisions regarding conservation, management and restoration of *O. acuminata*. Inferred from this case study, we discuss the perspectives on the use of plastome super-barcodes in plant species conservation.

## Materials and Methods

### Taxon Sampling

**FIGURE 1:**
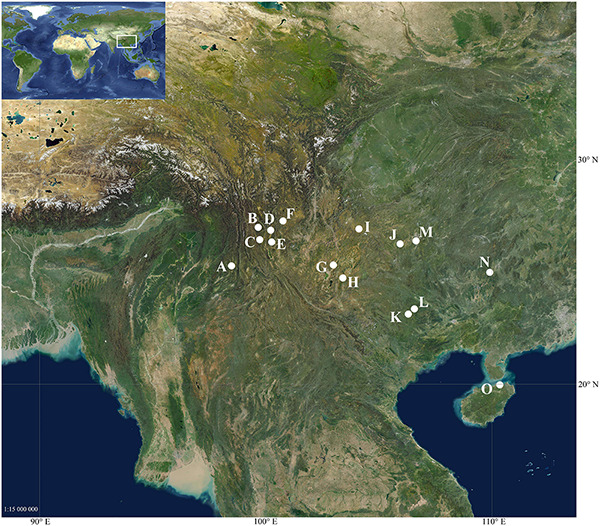
Geographic distribution of sampled populations. **(A)** Beihai Lake, Tengchong, Yunnan, China (25.276° N, 98.494° E); **(B)** Qixian Lake, Yulong, Yunnan China (26.979° N, 99.671° E); **(C)** Jian Lake, Jianchuan, Yunnan, China (26.436° N, 99.743° E); **(D)** *O.* Lashihai Lake, Lijiang, Yunnan, China (26.855° N, 100.226° E); **(E)** Heilongtan, Heqing, Yunnan, China (26.334° N, 100.270° E); **(F)** Lugu Lake, Ninglang, Yunnan, China (27.263° N, 100.770° E); **(G)** Heilongtan, Songming, Yunnan, China (25.314° N, 102.998° E); **(H)** Changhu Lake, Shilin, Yunnan, China (24.744° N, 103.417° E); **(I)** Caohai Lake, Weining, Guizhou, China (26.910° N, 104.129° E); **(J)** Anshun, Guizhou, China (26.253° N, 105.948° E); **(K)** Equan River, Jingxi, Guangxi; China (23.146° N, 106.311° E); **(L)** Longtan River, Debao, Guangxi, China (23.379° N, 106.583° E); **(M)** Huaxi, Guiyang, Guizhou, China (26.384° N, 106.654° E); **(N)** Baishou River, Yongfu, Guangxi, China (24.993° N, 109.907° E); **(O)** Haikou, Hainan, China (20.018° N, 110.349° E).

Among the six phenotypic varieties of *O. acuminata*, *O. acuminata* var. *tonghaiensis* was not found after many field explorations within the areas of known distribution and is most likely extinct. For the remaining varieties, our field collections approximately cover all known populations. According to the phylogeny recovered by Li et al. (2020c), we also included *O. alismoides*, *O. balansae*, and *O. guanyangensis* in phylogenetic and species delimitation analyses to investigate their relationships to *O. acuminata*, and to explore taxonomic boundaries among these taxa. In total 60 accessions (Figure 1 and Table 1), representing *O. acuminata* var. *acuminata* (18 accessions from 3 populations), *O. acuminata* var. *crispa* (8 accessions from 1 populations), *O. acuminata* var. *jingxiensis* (15 accessions from 3 populations), *O. acuminata* var. *lunanensis* (3 accessions from 1 populations), *O. acuminata* var. *songmingensis* (2 accessions from 1 population), *O. alismoides* (2 accessions from 1 populations), *O. balansae* (9 accessions from 4 populations), and *O. guanyangensis* (3 accessions from 1 populations) were sampled from wild populations. The sampling size of these taxa was determined according to their distribution range and population size. For those taxa possessing narrowly restricted distribution and extremely small population size, such as *O. acuminata* var. *lunanensis*, *O. acuminata* var. *songmingensis*, and *O. guanyangensis*, we tried to sample at least two individuals per taxon. The extensive sampling strategy adopted in this study allows a robust test for the current taxonomic designation of the target taxa.

**TABLE 1:** Samples used in this study with population, voucher, GenBank accessions, and plastome features.

Population*	**Taxa**	**Voucher**	**GenBank accessions**	**Plastome**		**LSC**		**IR**		**SSC**	
				**Size (bp)**	**GC (%)**	**Size (bp)**	**GC (%)**	**Size (bp)**	**GC (%)**	**Size (bp)**	**GC (%)**
A	*O. alismoides*	TC-M001	MW442046	157,882	36.60	87,703	34.30	25,557	43.10	19,065	29.60
		TC-M007	MW442003	157,881	36.60	87,702	34.30	25,557	43.10	19,065	29.60
B	*O. balansae*	YL-A004	MW442054	156,885	36.60	87,372	34.40	25,063	43.20	19,387	30.00
		YL-A005	MW442044	156,895	36.60	87,552	34.30	24,978	43.30	19,387	30.00
		YL-A008	MW442027	156,894	36.60	87,381	34.40	25,063	43.20	19,387	30.00
C	*O. acuminata* var. *acuminata*	JC-C003	MW442045	157,012	36.60	87,489	34.30	25,050	43.20	19,423	29.90
		JC-C004	MW442015	157,032	36.60	87,534	34.30	25,051	43.20	19,396	29.90
		JC-C005	MW442031	157,013	36.60	87,490	34.30	25,050	43.20	19,423	29.90
		JC-C006	MW442029	157,037	36.60	87,522	34.30	25,051	43.20	19,413	29.90
		JC-C007	MW442058	157,030	36.60	87,702	34.30	24,966	43.30	19,396	29.90
		JC-C009	MW441999	157,004	36.60	87,483	34.30	25,050	43.20	19,421	29.90
		JC-C010	MW442020	157,010	36.60	87,487	34.30	25,050	43.20	19,423	29.90
		JC-C013	MW442036	157,033	36.60	87,535	34.30	25,001	43.30	19,496	29.90
		JC-C017	MW442023	157,033	36.60	87,705	34.30	24,966	43.30	19,396	29.90
		JC-C019	MW442034	157,006	36.60	87,483	34.30	25,050	43.20	19,423	29.90
		JC-C020	MW442002	157,045	36.60	87,699	34.30	24,969	43.30	19,408	29.90
		JC-LYD	MW442013	157,007	36.60	87,487	34.30	24,945	43.30	19,420	29.90
D	*O. acuminata* var. *acuminata*	LJ	MW442051	156,920	36.60	87,418	34.40	25,058	43.20	19,386	29.90
E	*O. Acuminata* var. *acuminata*	HQ-B001	MW442025	156,977	36.60	87,478	34.40	25,058	43.20	19,383	29.90
		HQ-B003	MW442048	156,975	36.60	87,469	34.40	25,058	43.20	19,390	29.90
		HQ-B006	MW442028	156,960	36.60	87,463	34.40	25,058	43.20	19,381	30.00
		HQ-B008	MW442059	156,949	36.60	87,454	34.40	25,058	43.20	19,379	29.90
		HQ-B011	MW442008	156,972	36.60	87,471	34.40	25,058	43.20	19,385	29.90
F	*O. acuminata* var. *crispa*	NL-E001	MW442039	156,983	36.60	87,653	34.30	24,973	43.30	19,384	30.00
		NL-E002	MW442049	156,982	36.60	87,482	34.30	25,058	43.20	19,384	30.00
		NL-E004	MW442057	156,981	36.60	87,481	34.30	25,058	43.20	19,384	30.00
		NL-E005	MW442024	156,982	36.60	87,482	34.30	25,058	43.20	19,384	30.00
		NL-E009	MW442000	156,980	36.60	87,480	34.30	25,058	43.20	19,384	30.00
		NL-E010	MW442035	156,981	36.60	87,481	34.30	25,058	43.20	19,384	30.00
		NL-E014	MW442026	156,980	36.60	87,480	34.30	25,058	43.20	19,384	30.00
		NL-E019	MW442021	156,981	36.60	87,650	34.30	24,973	43.30	19,385	30.00
G	*O. acuminata* var. *songmingensis*	SM-D006	MW442041	157,646	36.60	87,645	34.30	25,557	43.10	18,887	29.90
		SM-D012	MW442053	157,647	36.60	87,816	34.30	25,472	43.20	18,887	29.90
H	*O. acuminata* var. *lunanensis*	SL-L004	MW442030	156,908	36.60	87,583	34.30	24,973	43.30	19,379	29.90
		SL-L015	MW442033	156,907	36.60	87,412	34.30	25,058	43.20	19,379	29.90
		SL-L019	MW442052	156,906	36.60	87,411	34.30	25,058	43.20	19,379	29.90
I	*O. balansae*	WN-F005	MW442009	156,941	36.60	87,437	34.30	25,057	43.20	19,390	29.90
		WN-F006	MW442007	156,920	36.60	87,416	34.30	25,057	43.20	19,390	29.90
		WN-F008	MW442019	156,918	36.60	87,414	34.30	25,057	43.20	19,390	29.90
		WN-F015	MW442022	156,922	36.60	87,588	34.30	24,972	43.30	19,390	29.90
J	*O. balansae*	AS-XJX	MW442038	156,982	36.60	87,396	34.30	25,078	43.20	19,430	29.90
K	*O. acuminata* var. *jingxiensis*	JX-I005	MW442012	156,861	36.60	87,418	34.30	24,988	43.30	19,467	29.90
		JX-I007	MW442011	156,861	36.60	87,418	34.30	24,988	43.30	19,467	29.90
		JX-I008	MW442040	156,862	36.60	87,589	34.30	24,953	43.30	19,367	30.00
		JX-I011	MW442014	156,835	36.60	87,393	34.30	25,038	43.20	19,366	30.00
		JX-I012	MW442043	156,834	36.60	87,394	34.30	25,038	43.20	19,364	30.00
		JX-I016	MW442032	156,863	36.60	87,590	34.30	24,953	43.30	19,367	30.00
		JX-I020	MW442055	156,861	36.60	87,588	34.30	24,953	43.30	19,367	30.00
		JX-J002	MW442047	156,850	36.60	87,405	34.30	25,038	43.20	19,369	30.00
		JX-J003	MW442056	156,832	36.60	87,391	34.30	25,038	43.20	19,365	30.00
		JX-J004	MW442005	156,832	36.60	87,561	34.30	24,953	43.30	19,365	30.00
		JX-J011	MW442017	156,832	36.60	87,391	34.30	25,038	43.20	19,365	30.00
L	*O. acuminata* var. *jingxiensis*	DB-G002	MW442018	156,816	36.60	87,541	34.30	24,954	43.30	19,367	29.90
		DB-H004	MW442042	156,814	36.60	87,368	34.30	25,038	43.20	19,370	30.00
		DB-H012	MW442006	156,761	36.60	87,320	34.40	25,038	43.20	19,365	30.00
		DB-H019	MW442001	156,761	36.60	87,490	34.30	24,953	43.30	19,365	30.00
M	*O. balansae*	GY-GYHX	MW442050	156,945	36.60	87,364	34.30	25,078	43.20	19,425	29.90
N	*O. guanyangensis*	YF-K005	MW442004	157,389	36.70	87,268	34.40	25,564	43.10	18,993	29.70
		YF-K006	MW442037	157,388	36.70	87,438	34.40	25,479	43.10	18,992	29.70
		YF-K009	MW442010	157,389	36.70	87,269	34.40	25,564	43.10	18,992	29.70
O	*O. cordata*	HK	MW442016	157,886	36.60	87,685	34.30	25,552	43.10	19,097	29.50

*Locality of each population is shown in Figure 1.

### Shotgun Sequencing, Plastome Assembly and Annotation

Total genomic DNA for each accession was isolated from ∼20 mg silica gel dried leaf tissues using the cetyltrimethylammonium bromide method of Doyle and Doyle (1987). Approximately 5 μg of purified genomic DNA was used to construct paired-end libraries with a TruSeq DNA Sample Prep Kit (Illumina, Inc., San Diego, CA, United States) following the manufacturer’s instructions. Shotgun sequencing was performed on the Illumina HiSeq 2500 system with 2 × 150 reads. Raw reads were subjected to the NGS QC Toolkit (Patel and Jain, 2012) to remove adaptors and low-quality reads with the default parameters.

Using the filtered reads, *de novo* assembly of complete plastome was performed by NOVOPlasty v2.7.0 (Dierckxsens et al., 2017) with *k*–mer of 31, and using the large subunit of RuBisCO gene of *O. acuminata* (HM257638) as the seed for iterative extension of contigs to recover the whole plastome of each accession. The newly assembled plastomes were annotated with the Dual Organellar Genome Annotator database (Wyman et al., 2004). The annotation of protein-coding genes was further confirmed with a BLAST search against the NCBI protein database. Genes putatively annotated as transfer RNA (tRNA) were further verified by tRNAscan-SE 1.21 (Schattner et al., 2005) with default parameters. The boundary of the large-single copy (LSC), small-single copy (SSC), and inverted-repeat (IR) regions for each plastome were visually examined and manually adjusted with Geneious V10.2.3 (Kearse et al., 2012).

### Phylogenetic Analyses

Based on inferred phylogenetic trees, we tested whether *O. acuminata* and allied species (*O. alismoides*, *O. balansae*, and *O. guanyangensis*) are evolutionarily distinctive entities. The complete plastome sequences were aligned using MAFFT v7.450 (Katoh and Standley, 2013) with manual adjustment where necessary. Phylogenetic trees were reconstructed using both maximum likelihood (ML) and Bayesian inference (BI) methods. *Ottelia cordata*, the closest relative of the ingroup (Li et al., 2020c), was used to root the phylogenetic tree. The best-fit sequence substitution model for complete plastomes (GTR + G) was selected using MODELTEST v3.7 (Posada and Crandall, 1998) with the Akaike information criterion (Posada and Buckley, 2004).

Maximum likelihood analyses were performed using RAxML-HPC BlackBox v8.1.24 (Stamatakis, 2006). The best-scoring ML tree was generated with 1,000 bootstrap (BS) replicates to obtain branch support. BI reconstructions were conducted using MrBayes v3.2 (Ronquist and Huelsenbeck, 2003). Two independent Markov Chain Monte Carlo runs were performed with 1,000,000 generations, sampling every 100 generations. An initial 25% of the sampled trees were discarded as burn-in. Posterior probability (PP) values were computed based on the remaining trees. Stationarity was considered to be reached when the average standard deviation of the split frequencies was <0.01.

### Sequence-Based Species Delimitation

There has been an explosion in molecular-based species delimitation approaches over the past 15 years (e.g., Yang and Rannala, 2010; Ence and Carstens, 2011; Masters et al., 2011; Puillandre et al., 2012b; Rannala and Yang, 2013; Zhang et al., 2013). As revealed by previous studies, using different approaches simultaneously to delineate species boundary allows the methods to compensate for each other’s weaknesses (e.g., Hebert et al., 2003; Kekkonen and Hebert, 2014; Mutanen et al., 2015). Based on the alignments of complete plastomes sequences, we used two sequence-based species delimitation tools to estimate the number of species-like units among *O. acuminata* and related taxa. The first was a distance-based method, automatic barcode gap discovery (ABGD), which statistically infers the barcode gap from the sequence data and clusters sequences into putative species based on the pairwise distances among group of individuals (Puillandre et al., 2012a). The ABGD analyses were conducted on the online server^1^ using three different distance models (JC69, K2P, and *P*-distances) with default settings (Pmin = 0.001, Pmax = 0.1, Steps = 10, X = 1.5, and Nb bins = 20). All assignments for intraspecific divergence (P) values between 0.0001 and 0.0100 were recorded. Next, we used the tree-based methods: multi-rate Poisson tree processes model (mPTP; Kapli et al., 2017) to explore putative species boundaries of the target species. Based on the phylogenetic species concept, this method uses nucleotide substitutions along the branches of the phylogenetic tree to determine putative species boundaries under the assumption that the number of intraspecific substitutions is smaller than that of interspecific substitutions, with both substitutions following a distinct Poisson distribution (Zhang et al., 2013). The mPTP v0.2.3 algorithm (Kapli et al., 2017), an improvement to PTP (Zhang et al., 2013), was run on the web server^2^ with standard default settings, using the ML trees of complete plastome as inputs, because the branch lengths of ML tree represent number of sequence mutations.

## Results

### Shotgun Sequencing and Plastome Assembly

**FIGURE 2:**
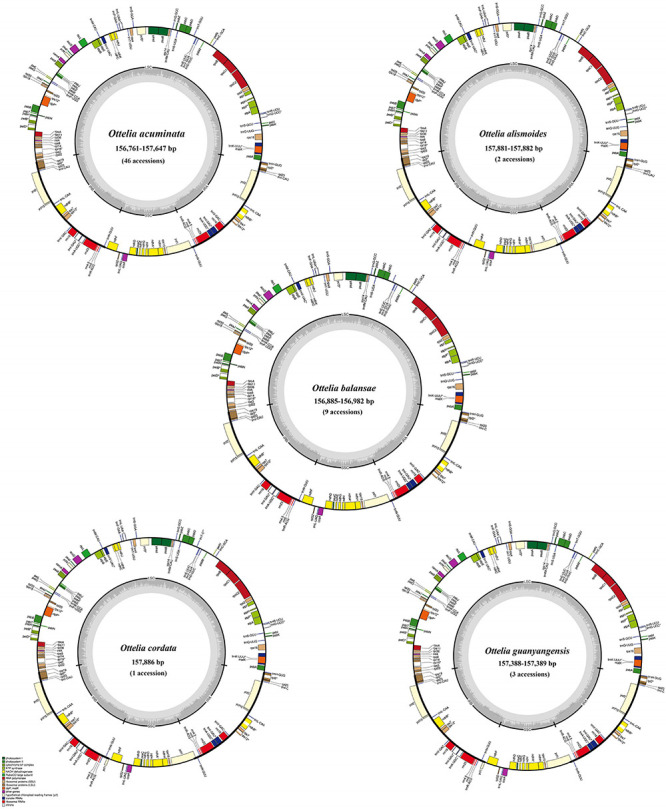
Map of *Ottelia* plastomes. Genes shown outside the circle are transcribed clockwise, and those inside are transcribed counterclockwise.

Based on cleaned shotgun reads, *de novo* assembly generated the complete plastome of all samples. These newly sequenced plastomes possess a typical quadripartite structure, with the sequence length varying from 156,761–157,886 bp, containing a pair of inverted repeats (IRs; 24,945–25,564 bp) separated by the LSC (87,268–87, 816 bp) and SSC (18,887–19,496 bp) regions (Figure 2 and Table 1). The gene content of each plastome includes 114 genes, including 79 protein-coding genes, 30 tRNA genes, and four plastid rRNA genes (Supplementary Table 1).

### Phylogenetic Reconstruction

**FIGURE 3:**
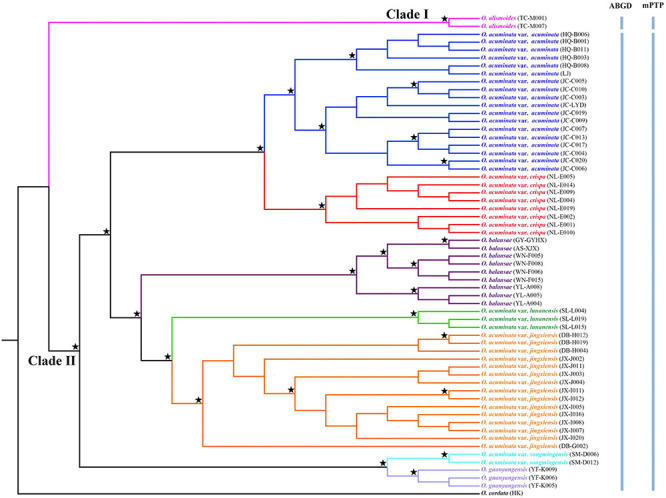
Phylogenetic relationships between *Ottelia acuminata* and closely related species based on Maximum likelihood (ML) and Bayesian inference (BI) of complete plastomes. Well-supported nodes (BS > 90% and PP > 0.95) are indicated with a star. Species delimitation schemes proposed by automatic barcode gap discovery (ABGD) and multi-rate Poisson tree processes model (mPTP) are reflected on the tree topology.

Alignment of the plastome sequences yielded a matrix of 159,782 positions, in which 1,818 variable sites (1.14%) were identified and 1,745 (1.09%) were parsimoniously informative. ML and BI analyses of complete plastomes produced identical tree topologies (Figure 3). Overall, all five varieties of *O. acuminata* (*O. acuminata* var. *acuminata*, *O. acuminata* var. *crispa*, *O. acuminata* var. *jingxiensis*, *O. acuminata* var. *lunanensis*, and *O. acuminata* var. *songmingensis*), as well as *O. alismoides*, *O. balansae*, and *O. guanyangensis* were resolved as well-supported monophyletic entities. The monophyly of *O. acuminata* as a single species, however, was not supported by either ML or BI phylogeny. Among the taxa, *O. alismoides* (Clade I) was sister to the clade that included the remaining taxa (Clade II). Within Clade II, *O. acuminata* var. *songmingensis* and *O. guanyangensis* formed the earliest diverging branch (BS = 100%, PP = 1.00), and the remaining taxa grouped into two well-supported subclades. The first one comprised accessions of *O. acuminata* var. *acuminata* and *O. acuminata* var. *crispa*. Within the second one, *O. acuminata* var. *jingxiensis* was sister to *O. acuminata* var. *lunanensis*, and these two taxa, in turn, were sister to *O. balansae*.

### Species Delimitation

Automatic barcode gap discovery and mPTP analyses of complete plastome sequences produced highly congruent results that are reflected in the inferred phylogenetic tree (Figure 3). The ABGD analyses (Table 2) resulted in a consistent count of species division (*n* = 2) with a range of prior intraspecific values (*P* = 0.0046–0.00836) using JC69, K2P, and *P*-distances with initial and recursive partitions. One putative species includes individuals of *O. alismoides*, while the other comprises individuals of *O. balansae*, *O. guanyangensis* and *O. acuminata* (Figure 3). The mPTP analyses yielded the same delimitation scheme as ABGD did: all individuals were grouped into two species-like units that coincide with the two putative species proposed by ABGD analyses (Figure 3), with both of them receiving high posterior support (PP = 1.00).

**TABLE 2:** The number of putative species recognized by Automatic Barcode Gap Discovery (ABGD) analyses of complete plastome sequences using three distance metrics.

**Subst. model**	**X**	**Partition**	**Prior intraspecific divergence (P)**			
			**0.001000**	**0.001668**	**0.002783**	**0.004642**
P	1.5	Initial	2	2	2	2
		Recursive	2	2	2	2
JC69	1.5	Initial	2	2	2	2
		Recursive	2	2	2	2
K2P	1.5	Initial	2	2	2	2
		Recursive	2	2	2	2

*X: relative gap width; P: p-distance; JC69: Jukes-Cantor 69; K2P: Kimura 2-parameter.*

## Discussion

### Taxonomic Delimitation of *Ottelia acuminata* and Conspecific Varieties

In this study, a sampling strategy that included multiple individuals within a species (or variety) representing different localities (if any) was employed to test for the evolutionary distinctiveness of each taxon. Additionally, two independent delimitation methods (mPTP and ABGD) were used to explore species delimitation, which allows the methods to compensate for weaknesses of the other method to develop a robust taxonomic delimitation framework (e.g., Hebert et al., 2003; Kekkonen and Hebert, 2014; Mutanen et al., 2015). Similar to an earlier study that did not include multiple individuals per taxon in a phylogenetic context (Li et al., 2020c), our plastome-based phylogeny failed to resolve *O. acuminata* as a monophyletic unit given two congeneric relatives (*O. balansae* and *O. guanyangensis*) were embedded within *O. acuminata* in the tree topology. This implies that the three species may have been incompletely separated from each other, thus recognizing them as distinct species may not hold (de Queiroz, 1998, 2007). This inference is justified by the results of the species delimitation analyses. Specifically, the ABGD analyses partitioned all the samples into two species-level entities corresponding to the two clades recovered by phylogenetic analyses, suggesting that Clade I (*O. alismoides*) and Clade II (*O. acuminata* + *O. balansae* + *O. guanyangensis*) represent two distinct species with significant genetic gaps between them (Puillandre et al., 2012b). Moreover, the mPTP analyses grouped all accessions into two putative species with high delimitation posterior probability, coinciding with the results found in the ABGD analyses. These reciprocal reinforcing results suggest that only two species-level taxonomic units that, respectively, correspond to the *O. alismoides* clade and the *O. acuminata* + *O. balansae* + *O. guanyangensis* clade can be recognized.

A previous study (Li et al., 2020c) suggests that the divergence between *O. alismoides* and the *O. acuminata* + *O. balansae* + *O. guanyangensis* clade occurred at ∼6.01 million years ago (Mya). The long-term differentiation developed a high degree evolutionary independence between them. As shown by phylogenetic analysis, they are two completely separated lineages in the tree topologies. Given that they reflect the unity of morphological uniqueness, genetic distinctiveness, and evolutionary independence, it is reasonable to recognized them as distinct species under the unified species concept (de Queiroz, 1998, 2007). Comparatively, it is indicated that the stem age of the *O. acuminata* + *O. balansae* + *O. guanyangensis* clade are more recent at ∼3.88 Mya (Li et al., 2020c). This implies that these taxa may have incompletely separated from each other due to their relatively short evolutionary histories. The speculation is justified by our phylogenetic analyses and sequence-based species delimitation schemes, which consistently indicate that *O. balansae* and *O. guanyangensis* are neither evolutionarily nor genetically distinct from *O. acuminata*. Under the updated concept of subspecies that recognizes incompletely separated lineages within a more inclusive lineage as subspecific taxa (de Queiroz, 2020), it is reasonable to reduce *O. balansae* and *O. guanyangensis* as conspecific varieties of *O. acuminata*.

On the other hand, *O. balansae* and *O. guanyangensis* share high levels of similarity in leaf, spathe, sepal, petal, and fruit morphologies with *O. acuminata*, but differ from the latter species in having bisexual flowers (versus unisexual flowers in *O. acuminata*) (Wang et al., 2010; Liu et al., 2018). However, it has been reported that there are scattered individuals with bisexual flowers in natural *O. acuminata* populations (Li, 1981; Jiang et al., 2010), and our field observations found that most individuals of *O. balansae* and *O. guanyangensis* produce unisexual flowers and only few individuals have bisexual flowers in wild populations. This suggests that the diagnostic character (bisexual versus unisexual flowers) used to distinguish *O. balansae* and *O. guanyangensis* from *O. acuminata* can be a plastic trait. Therefore, prior morphology-based taxonomic studies overemphasized intraspecific morphological differences to establish species, thus leading to taxonomic over-splitting of species. As a result, it is reasonable to merge *O. balansae* and *O. guanyangensis* into *O. acuminata.*

The lack of continuous water systems among lakes, ponds, and rivers in southwest China, which severely restricted pollen and seed dispersal among fragmented populations, may have led to significant isolation events in *O. acuminata* (Zhang et al., 2009; Long et al., 2010; Chen et al., 2017; Guo et al., 2019). The limited gene flow among isolated populations would result in significant genetic differentiations in *O. acuminata*, and thus triggered the formation of diverse conspecific varieties (Chen et al., 2017). Although there is no significant morphological difference between these phenotypic varieties (Li et al., 2020c), they were identically resolved as well-supported monophyletic units by our complete plastome-based phylogenies. This suggests the genetic boundaries among these taxa are large enough to ensure accurate varietal identification using complete plastome DNA sequences as super-barcodes. Nevertheless, previous studies revealed that these varieties identically harbor low genetic diversity and weak population genetic differentiation (Zhang et al., 2009; Long et al., 2010; Chen et al., 2017; Zhai et al., 2018; Guo et al., 2019). Moreover, except for *O. acuminata* var. *acuminata*, *O. acuminata* var. *jingxiensis*, and *O. balansae*, the remaining taxa occur in only a single population and possess extremely small population size (Li, 1985; Jiang et al., 2005, 2010; Wang et al., 2010). From this perspective, they are more likely to represent genetically differentiated and geographically isolated intraspecific populations than separately evolving metapopulation lineages. Taken together, these findings provide good support to our taxonomic proposal that recognizes *O. acuminata* var. *acuminata*, *O. acuminata* var. *crispa*, *O. acuminata* var. *jingxiensis*, *O. acuminata* var. *lunanensis*, and *O. acuminata* var. *songmingensis* as distinct varieties, and reduces *O. balansae* and *O. guanyangensis* as conspecific varieties of *O. acuminata*.

### Conservation Implications

Aquatic macrophytes are non-negligible targets for conservation management since many species have become locally or even globally extinct during the past decades due to water pollution, eutrophication, changes in hydrological regime, and biological invasion (Sand-Jensen et al., 2000; Phillips et al., 2016; Zhang et al., 2017). Credible delineation of species boundaries is an essential step in species conservation (Hopkins and Freckleton, 2002; Sites and Marshall, 2003; Mace, 2004). It is generally accepted that well delimited species are fundamental to allow adequate conservation and biodiversity management (Mace, 2004). Contrarily, poor delineation of species boundaries usually makes it difficult to properly address conservation issues. For instance, species delimitations that are too broad will result in the underestimation of true species richness and improper assignation of conservation priorities because some threatened species are overlooked (Rojas, 1992; Sites and Crandall, 1997; Prance et al., 2000; Mace, 2004), while taxonomic over-splitting of species may lead to the misallocation of limited conservation resource to widespread species that are not at risk of extinction (Agapow et al., 2004; Mace, 2004; Joppa et al., 2011). In this study, analyses of plastome super-barcodes develop a clear-cut taxonomic delimitation of *O. acuminata* and its conspecific varieties, which will help to better inform future decisions regarding the conservation and management of this severely threatened submerged macrophyte.

Based on our data, several strategies can be proposed for the conservation and restoration of *O. acuminata*. Although the species as a whole is not at a high risk of extinction due to its relatively wide distribution range and large population size, it is highly relevant for conservation since the conspecific varieties possess high level of evolutionary and genetic distinctiveness with mostly small distributions. The preservation of genetic diversity and evolutionary potential is a primary goal for the conservation of threatened species (Milligan et al., 1994; Margules and Pressey, 2000). The conservation strategy for *O. acuminata* should be aimed at preserving all its conspecific varieties because they represent an indispensable evolutionary legacy. Among them, *O. acuminata* var. *crispa*, *O. acuminata* var. *lunanensis*, and *O. acuminata* var. *songmingensis* are recommended here as the prioritized taxa for conservation, because they possess a particularly narrow distribution and occur in only one lake or pond (Li, 1985; Jiang et al., 2005, 2010; Wang et al., 2010) thus are more vulnerable to environmental changes and anthropogenic disturbances (Mckinney, 1997; Henle et al., 2004).

Extant populations of *O. acuminata* are facing severe threats, such as habitat degradation, anthropogenic disturbances, and introduction of herbivorous fish (Li, 1985, 1988; Godo et al., 2003; Liang and Li, 2007; Jiang et al., 2010; Yang et al., 2012). In addition to *in situ* conservation, it is necessary to establish full-scale germplasm collections for *ex situ* conservation (Heywood and Iriondo, 2003). Due to the significant evolutionary and genetic distinctiveness between *O. acuminata* varieties, their germplasms need to be separately collected and propagated to prevent mixing divergent gene pools. Moreover, for restoration of natural *O. acuminata* populations, the reciprocal introduction and reintroduction between different varieties should be avoided, as this action may lead to outbreeding that may change the gene pool of locally adapted populations (Fischer and Lindenmayer, 2000; Edmands, 2007). Therefore, there is considerable need to identify *O. acuminata* varieties and to characterize their germplasms for conservation purposes. Our data show that the use of plastome super-barcodes meet this requirement, thus serving as a useful tool for proper conservation, management, and restoration of *O. acuminata*.

### Perspectives on the use of Plastome Super-Barcodes in Plant Species Conservation

Taxonomy and biodiversity conservation are interdependent practices (Mace, 2004). On global and regional scales, a taxonomic diversity inventory and estimates of the number of species that are under threat are essential for managing and conserving biodiversity (May, 1988; May and Beverton, 1990; Margules and Pressey, 2000; Mace et al., 2003). Recently, DNA barcoding has been widely used as a rapid and cost-effective tool for biodiversity inventory and for monitoring and assessment of threatened species (reviewed by Kress et al., 2015; Hollingsworth et al., 2016; Wilson et al., 2016). Nevertheless, the use of standard DNA barcodes may result in biased estimates of species diversity and ambiguous species identification due to insufficient performance in discriminating plant species, especially in lineages that have experienced rapid radiations or complicated evolutionary histories (Hollingsworth et al., 2009, 2011, 2016; Hollingsworth, 2011; Coissac et al., 2016). As revealed by previous studies (e.g., Kane et al., 2012; Ruhsam et al., 2015; Firetti et al., 2017; Ji et al., 2019, 2020; Zhu et al., 2019; Li et al., 2020; Ślipiko et al., 2020) and our data, the use of plastome super-barcodes performs well in species identification and delimitation as well as in discovery of cryptic or overlooked diversity. This tool has great potential to lessen the challenges of biodiversity inventory and setting conservation priorities for threatened species.

Currently, complete plastomes of most plants can be easily obtained through a relatively low coverage shotgun sequencing of genomic DNA (Straub et al., 2012). Compared with Restriction-site Associated DNA sequencing (Baird et al., 2008), another recommended technique for plant barcoding 2.0 (Hollingsworth et al., 2016), a promising advantage of using plastome super-barcodes for species identification and delimitation is its universality (Kane et al., 2012). Additionally, with the advances of NGS technology, it has become much easier to generate complete plastome sequences even with using trace and highly degraded genomic DNA to prepare shotgun libraries (Zeng et al., 2018), making it feasible to catalog species diversity and to monitor threatened plants with herbarium specimens and other plant products. With the plastomes of a wide spectrum of plant lineages increasingly available in public database (e.g., NCBI GenBank), the plastome super-barcode reference libraries of known taxa are constantly enriched. We are optimistic that the plastome super-barcoding approach will likely to product more information of conservation values.

## Data Availability Statement

The datasets presented in this study can be found in online repositories. The names of the repository/repositories and accession number(s) can be found in the article/ Supplementary Material.

## Author Contributions

YJ and YZ conceived the research. YJ, JY, SW, and ZY collected and analyzed the data. YJ wrote the manuscript. JL discussed the results and revised the manuscript. All authors contributed to the article and approved the submitted version.

## Conflict of Interest

The authors declare that the research was conducted in the absence of any commercial or financial relationships that could be construed as a potential conflict of interest.

## Footnotes

1 https://bioinfo.mnhn.fr/abi/public/abgd/abgdweb.html

2 http://species.h-its.org/ptp/
