# Supplementary material for: Deciphering the Taxonomic Delimitation of Ottelia acuminata (Hydrocharitaceae) Using Complete Plastomes as Super-Barcodes
Source: Front Plant Sci. 2021 Jul 15;12:681270. doi: 10.3389/fpls.2021.681270 (PMC8320023; doi:10.3389/fpls.2021.681270)
Supplement: Supplementary file 1 [file Table_1.DOCX]

| Category of Genes | Group of gene | Name of gene |
| --- | --- | --- |
| Self-replication | Ribosomal RNA genes | *rrn*4.5×2, *rrn*5×2, *rrn*16×2, *rrn*23×2 |
|  | Transfer RNA genes | *trn*C-GCA, *trn*D-GUC, *trn*E-UUC, *trn*F-GAA, *trn*G-GCC, *trn*G-UCC***, *trn*H-GUG, *trn*K-UUU***, *trn*L-UAA***, *trn*L-UAG, *trn*M-CAU, *trn*P-UGG, *trn*Q-UUG, *trn*R-UCU, *trn*S-GCU, *trn*S-GGA, *trn*S-UGA, *trn*T-UGU, *trn*T-GGU, *trn*V-UAC***, *trn*Y-GUA, *trn*W-CCA, *trn*fM-CAU, *trn*A-UGC*×2, *trn*I-CAU*×*2, *trn*I-GAU*×2, *trn*L-CAA×2, *trn*N-GUU×2, *trn*R-ACG×2, *trn*V-GAC×2 |
|  | Ribosomal protein (small subunit) | *rps*2, *rps*3, *rps*4, *rps*7×2, *rps*8, *rps*11, *rps*12**×2, *rps*14, *rps*15, *rps*16*, *rps*18, *rps*19 |
|  | Ribosomal protein (large subunit) | *rpl*2×2, *rpl*14, *rpl*16***, *rpl*20, *rpl*22, *rpl*23×2, *rpl*32, *rpl*33, *rpl*36 |
|  | RNA polymerase | *rpo*A, *rpo*B, *rpo*C1***, *rpo*C2 |
|  | Translational initiation factor | *inf*A |
| Genes for photosynthesis | Subunits of photosystem I | *psa*A, *psa*B, *psa*C, *psa*I, *psa*J*, ycf*3**, *ycf*4 |
|  | Subunits of photosystem II | *psb*A, *psb*B, *psb*C, *psb*D, *psb*E, *psb*F, *psb*H, *psb*I, *psb*J, *psb*K, *psb*L, *psb*M, *psb*N, *psb*T, *psb*Z |
|  | Subunits of cytochrome | *pet*A*, pet*B***, *pet*D***, *pet*G, *pet*L, *pet*N |
|  | Subunits of ATP synthase | *atp*A, *atp*B, *atp*E, *atp*F*, *atp*H, *atp*I |
|  | Large subunit of Rubisco | *rbc*L |
|  | Subunits of NADH dehydrogenase | *ndh*A*, *ndh*B*×2, *ndh*C, *ndh*D, *ndh*E, *ndh*F, *ndh*G, *ndh*H, *ndh*I, *ndh*J, *ndh*K |
| Other genes | Maturase | *mat*K |
|  | Envelope membrane protein | *cem*A |
|  | Subunit of acetyl-CoA | *acc*D |
|  | Synthesis gene | *ccs*A |
|  | ATP-dependent protease | *clp*P**** |
|  | Component of TIC complex | *ycf*1×2 |
| Genes of unknown function | Conserved open reading frames | *ycf*2×2, *ycf*15^#^×2 |

**Table S1.** List of genes identified in *Ottelia* plastomes.

×2: Two gene copies in IR regions; *: With one intron; **: With two introns；^#^: Pseudogene
