# Supplementary material for: Deciphering the Taxonomic Delimitation of Ottelia acuminata (Hydrocharitaceae) Using Complete Plastomes as Super-Barcodes
Source: Front Plant Sci. 2021 Jul 15;12:681270. doi: 10.3389/fpls.2021.681270 (PMC8320023; doi:10.3389/fpls.2021.681270)
Supplement: Supplementary file 2 [file Table_2.DOCX]

**Table S2.** Sequence variation identified in the alignment of *Ottelia* plastomes.

| Sequence | Aligned length (bp) | No. of variable sites (divergence %) | No. of parsimony informative sites (divergence %) |
| --- | --- | --- | --- |
| Entire plastid genome | 159,782 | 1,818 (1.14) | 1,745 (1.09) |
